# Diagnostic Value of TLE1 in Synovial Sarcoma: A Systematic Review and Meta-Analysis

**DOI:** 10.1155/2020/7192347

**Published:** 2020-01-29

**Authors:** Marc El Beaino, Daniel C. Jupiter, Tarek Assi, Elie Rassy, Alexander J. Lazar, Dejka M. Araujo, Patrick P. Lin

**Affiliations:** ^1^Department of Orthopaedic Oncology, University of Texas MD Anderson Cancer Center, Houston, TX, USA; ^2^Department of Orthopaedic Surgery and Rehabilitation Medicine, State University of New York, Downstate Medical Center, Brooklyn, NY, USA; ^3^Department of Preventive Medicine and Community Health, University of Texas Medical Branch, Galveston, TX, USA; ^4^Department of Orthopaedic Surgery and Rehabilitation, University of Texas Medical Branch, Galveston, TX, USA; ^5^Department of Cancer Medicine, Gustave Roussy Institute, F-94805 Villejuif, France; ^6^Departments of Pathology & Genomic Medicine, University of Texas MD Anderson Cancer Center, Houston, TX, USA; ^7^Department of Sarcoma Medical Oncology, University of Texas MD Anderson Cancer Center, Houston, TX, USA

## Abstract

**Background:**

Synovial sarcoma can present morphologically in multiple forms, including biphasic and monophasic subtypes. As a result, the histological diagnosis can sometimes be challenging. Transducin-Like Enhancer 1 (TLE1) is a transcriptional corepressor that normally is involved in embryogenesis and hematopoiesis but is also expressed in certain tumors. This systematic review examines the potential role of TLE1 as a diagnostic biomarker for the synovial sarcoma. *Materials and Methods*. A literature review and meta-analysis were conducted using the electronic databases Pubmed, the Cochrane Library, and Google Scholar. Thirteen studies met our eligibility criteria and were selected for in-depth analysis.

**Results:**

The mean sensitivity and specificity of TLE1 in detecting synovial sarcoma were 94% (95% CI 91%–97%) and 81% (95% CI 72%–91%), respectively, when all studies were aggregated together. The mean positive predictive value (PPV) of TLE1 was 75% (95% CI 62%–87%), whereas the negative predictive value (NPV) was 96% (95% CI 93%–98%).

**Conclusion:**

TLE1 is a sensitive and specific marker for synovial sarcoma that can aid in its diagnosis. Due to its involvement in several relevant signaling pathways, TLE1 might have direct relevance to the pathophysiology of the disease.

## 1. Introduction

**Figure 1:**
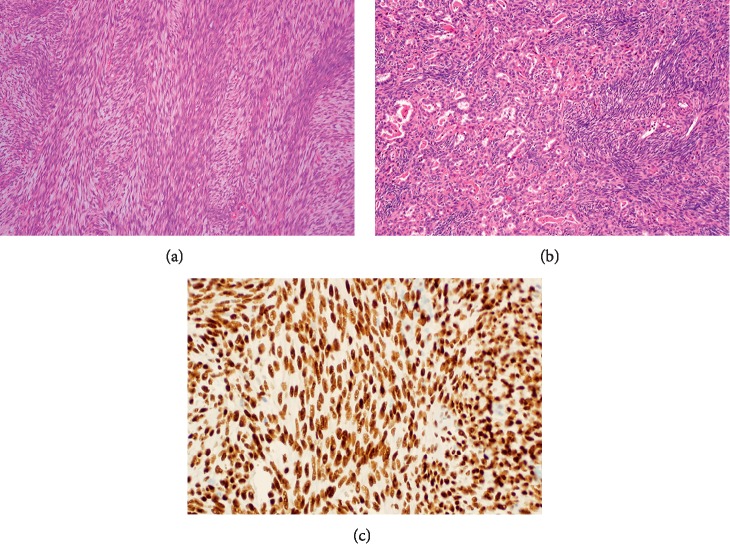
Histology of synovial sarcoma and immunohistochemical staining for TLE1. The common morphologic variants of synovial sarcoma are (a) monophasic spindle cell (hematoxylin and eosin stain, magnification 40x) and (b) biphasic with spindle cells and glandular differentiation (hematoxylin and eosin stain, magnification 40x). (c) Immunohistochemistry reveals nuclear staining with TLE1 in a monophasic synovial sarcoma.

Synovial sarcoma is a translocation-associated soft-tissue tumor that can arise at any age and in any anatomic location [1, 2]. It is driven by oncofusions involving the *SS18* gene on chromosome 18 with an *SSX* partner on chromosome X, frequently *SSX1* and *SSX2*, and rarely *SSX4* [3]. Synovial sarcoma can be morphologically classified into three main categories: the monophasic type predominantly composed of fascicles of spindle-shaped cells (Figure 1(a)), the biphasic subtype characterized by variable areas of spindle cells and glandular-like epithelium (Figure 1(b)), and poorly differentiated synovial sarcoma commonly including sheets of small blue round cells [4].

Diagnosis of synovial sarcoma is based on a combination of findings, including its characteristic morphology, immunohistochemical profile, and identification of the driver translocation [5]. Despite being the gold standard in establishing diagnosis, *SS18-SSX* detection can be challenging in rare cases, since some tumors (<2% of cases) can be driven by other less common cryptic and genetic rearrangements [6–8]. Another diagnostic challenge is the fact that several mesenchymal and nonmesenchymal neoplasms can exhibit morphological features similar to those of synovial sarcoma. The current immunohistochemical biomarkers used in such cases are valuable, but are limited by their specificities and sensitivities [9–11]. There is therefore a need to identify and develop new, reliable markers that can aid in the diagnosis of this tumor.

The Transducin-Like Enhancer (*TLE*) of split genes encode human transcriptional corepressors that are involved in embryogenesis and hematopoiesis [12, 13]. Gene expression profiling studies have consistently shown the *TLE* family of genes, *TLE1* in particular, to be overexpressed in the nuclei of synovial sarcoma cells [14, 15] (Figure 1(c)). Several immunohistochemical studies, involving whole-tissue sections or tissue microarrays, have analyzed the sensitivity and specificity of TLE1 in this disease [16–28]. Despite some inconsistent results, this marker seems to have notable utility in guiding pathologists in their differential diagnosis. We therefore sought to conduct a meta-analysis with the goal of assessing the value of TLE1 as a diagnostic marker for synovial sarcoma.

## 2. Materials and Methods

Pubmed, the Cochrane Library, and the Google Scholar databases (updated to May 2, 2019) were systematically searched for studies regarding the diagnostic value of TLE1 in synovial sarcoma. The search syntax used included the keywords “TLE1” OR “TLE-1” AND “synovial sarcoma,” and the search was restricted to English language and to human subject studies. Retrieved articles' titles and abstracts were examined and then checked for eligibility. The following inclusion criteria were used to identify studies for further analysis: (1) full-text publication evaluating TLE1 as a diagnostic biomarker in synovial sarcoma; (2) presented data including sample sizes of synovial and nonsynovial sarcomas samples; and (3) description of immunohistochemical methods used to detect and measure TLE1 expression. Conference abstracts, comments, and case reports were excluded, as were studies performed on cell lines rather than samples of suspected tumor.

All data were independently abstracted in duplicate by two investigators (MEB and TA) according to the inclusion criteria. Information retrieved from each publication included the first author's name, year of publication, antigen retrieval method (temperature, buffer, and pH), TLE1 antibody specifications (clonality, species, manufacturer, and dilution), number of cases of synovial sarcoma and mimics, histologic diagnosis, and grading system for TLE1 expression, as well as the sensitivity, specificity, positive, and negative predictive values of TLE1 for synovial sarcoma (or data from which these measure could be derived). Authors were contacted in case missing data were not reported in their respective articles.

Statistical analyses were performed using the metafor package within R (R Core Team, R Foundation for Statistical Computing, Vienna, Austria, https://www.r-project.org/) [29]. Sensitivity and specificity, as well as positive and negative predictive values were all computed with 95% confidence intervals (CI). Random effect models were used to account for interstudy variability, which was summarized with the *I*^2^ and *Q* statistics. Forest and funnel plots were drawn to summarize results and assess for systematic bias, respectively. Various sensitivity analyses were performed. First, we examined all studies. Next, we examined only studies that used either one of the two most commonly used immunohistochemical scoring methods and then separately examined studies using only one of those methods. We observed that one paper (by Chuang et al. [18]) presented results using both of these methods: we included the appropriate data from this paper that were applicable to our subanalyses.

## 3. Results

Based on their titles and abstracts, sixteen relevant citations evaluating TLE1 as a diagnostic marker in synovial sarcoma were identified in our literature query. Three articles were excluded from the subsequent analysis since they were non-English, did not include synovial sarcoma in their data, or were performed on synovial sarcoma cell lines [30–32]. The remaining thirteen publications were selected for further evaluation. After full-text reading, all thirteen articles met our eligibility criteria and were included in our meta-analysis. The included studies' characteristics are summarized in Table 1.

The mean sensitivity and specificity of TLE1 in detecting synovial sarcoma were 94% (95% CI 91%–97%) and 81% (95% CI 72%–91%), respectively, when all studies were aggregated together, regardless of the immunohistochemical grading system used to measure the marker's expression. The mean positive predictive value (PPV) of TLE1 was 75% (95% CI 62%–87%), whereas the negative predictive value (NPV) was 96% (95% CI 93%–98%). These values were similar to the ones computed in our subgroup analyses restricted to the grading systems 1 or 2, the two systems that were most commonly relied upon for TLE1 expression measurement (Table 2). However, all diagnostic metrics were slightly higher in the second system, when compared with the first.

**Figure 2:**
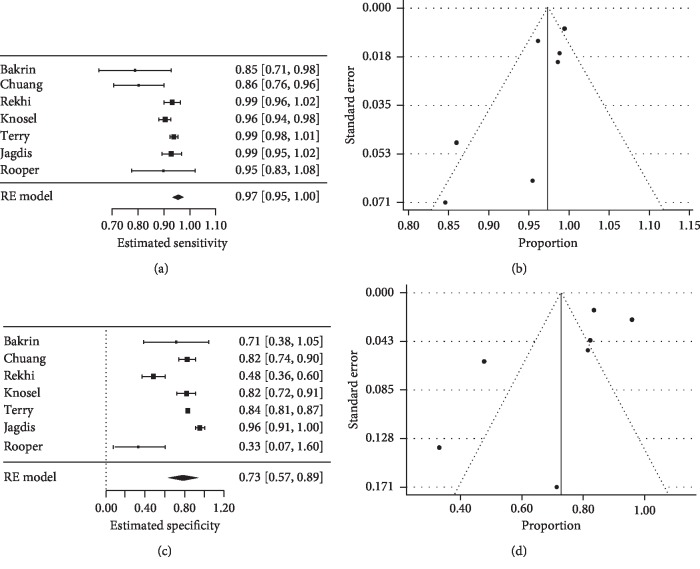
Sensitivity and specificity of TLE1 for synovial sarcoma. When limited to the studies that used a 2+ cut-off criterion as a positive TLE1 expression, forest plots detected a (a) sensitivity of 97% and a (c) specificity of 73%. Funnel plots were drawn to assess the systematic bias regarding both the sensitivity (b) and the specificity (d) of TLE1 in synovial sarcoma.

Seven studies used a cut-off of 2+ to consider a TLE1 expression positive, regardless of the immunohistochemical grading system [17, 18, 21, 22, 25–27]. This prompted us to utilize this criterion to perform another subanalysis. In such cases, TLE1 sensitivity increased to 97% (95% CI 95%–100%), while its specificity decreased to 73% (95% CI 57%–89%) (Figure 2). The PPV and NPV remained similar to what we had already seen, with values of 74% (95% CI 58%–89%) and 94% (95% CI 88%–100%), respectively.

## 4. Discussion

Synovial sarcoma is a soft-tissue malignancy that can be histologically classified into three different subtypes: (1) monophasic, composed of spindle-shaped cells; (2) biphasic, encompassing spindle-shaped cells and glandular-like areas; and (3) poorly differentiated, characterized mainly by sheets of small round cells [4]. The diagnosis of synovial sarcoma is not always straightforward in that the monophasic and poorly differentiated subtypes can sometimes be very difficult to distinguish from other mesenchymal and nonmesenchymal tumors, such as fibrosarcoma or undifferentiated pleomorphic sarcomas. Biomarkers that are being currently used in clinical practice to guide pathologists in the differential diagnosis have limited diagnostic value [9–11].

The majority of synovial sarcomas are driven by an *SS18-SSX* translocation, which fuses the *SS18* gene on chromosome 18 with an *SSX* gene on chromosome X. The sensitivity and specificity of this oncoprotein in detecting synovial sarcoma have been established, which makes its detection crucial to establish the diagnosis [3, 33]. Nine *SSX* genes (*SSX1*–*9*) have been identified to date, but the contribution of each to synovial sarcoma remains unclear [34]. The most common *SS18* fusion partners include *SSX1*, *SSX2*, and rarely *SSX4*. However, some tumors may not exhibit the pathognomonic translocation, and may therefore stem from alternative genetic or cryptic anomalies [6–8]. Such cases underscore the need for a robust array of diagnostic tools capable of confirming the diagnosis of synovial sarcoma while ruling out other potentially confounding tumors.

*TLE1* is a member of the *TLE* family of genes that encode Groucho-like transcriptional corepressors and is one of the most frequently overexpressed genes in synovial sarcoma [14, 15, 35, 36]. TLE1 binds other basic helix-loop-helix (bHLH) proteins to repress target genes [37–39]. Of these target genes, T-Cell Factor/Lymphoid Enhancer Factor (TCF/LEF), Hairy and Enhancer of Split (HES), and Homeobox proteins (NKX) constitute key mediators of the Wnt/*β*-catenin, Notch, and Sonic Hedgehog pathways, respectively. Several other genes pertaining to these networks may be dysregulated in synovial sarcoma, including *LEF1*, *AXIN2*, *TCF7*, and *FZD* homologues in the Wnt/*β*-catenin, *HES1*, and *NOTCH1* in the Notch, as well as *SMO* and *PTCH* in the Sonic Hedgehog pathways [15, 36, 40, 41]. By competing with and dislodging *β*-catenin and other coactivators from their respective transcriptional activator complexes, *TLE1* interacts with bHLH corepressor proteins to generate inhibitor entities that suppress gene transcription [37–39, 42]. Moreover, it alters histone deacetylase activity, which has been shown to be relevant in synovial sarcoma and alters epigenetic signals through transcriptional mediators [43–46]. For example, the Activating Transcription Factor 2 (ATF2) binds to SS18-SSX and redirects it to ATF2-related promoters to induce their corresponding genes' transcription; however, in the presence of TLE1, SS18-SSX will be displaced and ATF2 target genes repressed [47].

In spite of the variable data regarding the diagnostic value of TLE1 in synovial sarcoma, our analysis indicates that the preponderance of evidence favors TLE1 as a legitimate biomarker for this disease. Due to its high sensitivity and specificity, absence or presence of TLE1 on histology will aid in excluding or confirming the diagnosis of synovial sarcoma. Its high positive and negative predictive values are also beneficial attributes since synovial sarcoma is a rare disease. In particular, our study supports the experience of many clinical laboratories of pathology in that the degree of nuclear reactivity affects the interpretation of this assay. Of note, though TLE1 alone is not sufficient for diagnosis of synovial sarcoma since it is present in other tumors, particularly peripheral nerve sheath tumors that would include schwannomas (100%), neurofibromas (30%), and malignant peripheral nerve sheath tumor (30%) [23], these entities can usually be distinguished from synovial sarcoma on the basis of morphology and other criteria.

There is a number of limitations to our analysis. The lack of a consistent grading system for immunohistochemical staining to quantify TLE1 expression made it difficult to compare results across studies. Several studies did not report data regarding the synovial sarcoma subtypes that were analyzed [18, 20, 22, 23, 25–28]. Others lacked details about the control group to which synovial sarcomas were compared [17, 28]. A particular study compared TLE1 and NKX2.2 expression only in synovial and Ewing sarcomas [26]. Some authors used multiple antibodies to detect TLE1, while others used different techniques in the same study [21, 27]. Terry et al. used both a TLE rat monoclonal and a TLE1 rabbit polyclonal antibody in their analysis [27]. Jagdis et al. reported their combined experience at two different institutions, in which one group used a cold antigen retrieval method, whereas the group at the other institution relied on a heat-induced antigen retrieval technique [21]. Nevertheless, our subgroup analysis did not reveal any substantive differences between the groups of studies relying on different histological systems; this justifies our including all retrieved articles in the same analysis. Based upon our analysis of the different sensitivities and specificities of the various scoring systems, we recommend using a 2+ cut-off to qualify as positive TLE1 staining and Grading System 2 (see Tables 1 and 2).

In conclusion, TLE1 is a sensitive and specific marker for synovial sarcoma that can aid in its diagnosis. Due to its involvement in several relevant signaling pathways in this disease, TLE1 might have direct relevance to the pathophysiology of the disease. Identification of the networks in which TLE1 is involved, from a molecular standpoint, may help develop further diagnostic tests and novel targeted therapies for synovial sarcoma.

## Figures and Tables

**Table 1:** Eligible studies' characteristics.

#	Year	Antibody type	Dilution	Buffer	pH	*T* (°C)	Grading system^*∗*^	SS sample	NSS sample	Entire sample
[16]	2015	Rabbit polyclonal	1 : 200	PBS	7.2	NR	1	74	72	146
[17]	2016	Mouse polyclonal	1 : 150	Citrate	6	95	2	26	7	33
[18]	2013	Rabbit polyclonal	1 : 25	EDTA	9	95	1, 2, 4	50	85	135
[19]	2011	Rabbit polyclonal	1 : 400	Citrate	6	97	3	73	139	212
[20]	2016	Rabbit polyclonal	1 : 100	EDTA	6	95	2	62	322	384
[21]	2009	Rabbit polyclonal	1 : 20	EDTA	8.4	ND	2	35	73	108
[22]	2010	Rabbit polyclonal	1 : 100	EDTA	8.4	95	4	259	60	319
[23]	2009	Rabbit polyclonal	1 : 100	EDTA	8	97	1	20	143	163
[24]	2011	Rabbit polyclonal	1 : 20	EDTA	8	95	6	15	25	40
[25]	2012	Rabbit polyclonal	1 : 250	EDTA	8	97	1	42	69	111
[26]	2017	Mouse monoclonal Rabbit polyclonal	NR	Citrate	NR	92	7	10	12	22
[27]	2007	Rabbit polyclonal Rat monoclonal	1 : 20 1 : 2	EDTA	8	95	2	94	602	696
[28]	2013	Rabbit polyclonal	1 : 100	EDTA	6	95	5	5	37	42

Abbreviations: SS: synovial sarcoma; NSS: nonsynovial sarcoma; PBS: phosphate-buffered saline; EDTA: ethylenediaminetetraacetic acid; NR: not reported; ND: no details. ^*∗*^Grading system 1: 0 (<5% of cells positive); 1 (5–25% of cells positive); 2 (25–50% of cells positive); 3 (>50% of cells positive); Grading system 2: 0 (staining not visible); 1 (<26% of cells positive); 2 (26–50% of cells positive at 40x or >50% at 100x objectives); 3 (>50% of cells positive at 40x); Grading system 3: 0 (staining not visible); 1 (<5% of cells positive); 2 (6–25% of cells positive); 3 (26–50% of cells positive); 4 (51–75% of cells positive); 5 (>75% of cells positive); Grading system 4: 0 (no staining); 1 (<10% of cells positive); 2 (11–50% of cells positive); 3 (51–80% of cells positive); 4 (>80% of cells positive); Grading system 5: >20% of cells positive; Grading system 6: 0 (negative); 1 (weak); 2 (moderate); 3 (strong); Grading system 7: 0 (<5% of cells positive); 1 (5–25% of cells positive); 2 (25–50% of cells positive); 3 (50–75% of cells positive); 4 (75–90% of cells positive); 5 (>90% of cells positive).

**Table 2:** TLE1 diagnostic metrics in our subgroup analyses.

	Sensitivity	Specificity	PPV	NPV
System 1	92% (95% CI 83%–100%)	74% (95% CI 53%–95%)	64% (95% CI 34%–93%)	96% (95% CI 92%–100%)
System 2	95% (95% CI 89%–100%)	88% (95% CI 82%–94%)	74% (95% CI 58%–91%)	99% (95% CI 98%–100%)
